# Iodine maps derived from sparse-view kV-switching dual-energy CT equipped with a deep learning reconstruction for diagnosis of hepatocellular carcinoma

**DOI:** 10.1038/s41598-023-30460-y

**Published:** 2023-03-03

**Authors:** Keigo Narita, Yuko Nakamura, Toru Higaki, Shota Kondo, Yukiko Honda, Ikuo Kawashita, Hidenori Mitani, Wataru Fukumoto, Chihiro Tani, Keigo Chosa, Fuminari Tatsugami, Kazuo Awai

**Affiliations:** 1 Diagnostic Radiology, Hiroshima University, 1-2-3 Kasumi, Minami-ku, Hiroshima, 734-8551 Japan; 2 Graduate School of Advanced Science and Engineering, Hiroshima University, 1-4-1 Kagamiyama, Higashi-Hiroshima, Hiroshima 739-8527 Japan

**Keywords:** Gastroenterology, Medical research

## Abstract

Deep learning-based spectral CT imaging (DL-SCTI) is a novel type of fast kilovolt-switching dual-energy CT equipped with a cascaded deep-learning reconstruction which completes the views missing in the sinogram space and improves the image quality in the image space because it uses deep convolutional neural networks trained on fully sampled dual-energy data acquired via dual kV rotations. We investigated the clinical utility of iodine maps generated from DL-SCTI scans for assessing hepatocellular carcinoma (HCC). In the clinical study, dynamic DL-SCTI scans (tube voltage 135 and 80 kV) were acquired in 52 patients with hypervascular HCCs whose vascularity was confirmed by CT during hepatic arteriography. Virtual monochromatic 70 keV images served as the reference images. Iodine maps were reconstructed using three-material decomposition (fat, healthy liver tissue, iodine). A radiologist calculated the contrast-to-noise ratio (CNR) during the hepatic arterial phase (CNR_a_) and the equilibrium phase (CNR_e_). In the phantom study, DL-SCTI scans (tube voltage 135 and 80 kV) were acquired to assess the accuracy of iodine maps; the iodine concentration was known. The CNR_a_ was significantly higher on the iodine maps than on 70 keV images (*p* < 0.01). The CNR_e_ was significantly higher on 70 keV images than on iodine maps (*p* < 0.01). The estimated iodine concentration derived from DL-SCTI scans in the phantom study was highly correlated with the known iodine concentration. It was underestimated in small-diameter modules and in large-diameter modules with an iodine concentration of less than 2.0 mgI/ml. Iodine maps generated from DL-SCTI scans can improve the CNR for HCCs during hepatic arterial phase but not during equilibrium phase in comparison with virtual monochromatic 70 keV images. Also, when the lesion is small or the iodine concentration is low, iodine quantification may result in underestimation.

## Introduction

The American Association for the Study of Liver Diseases recommends dynamic computed tomography (CT) or magnetic resonance imaging (MRI) for diagnosis and response assessment of patients with hepatocellular carcinoma (HCC)^1–3^. Most malignant tumors are diagnosed pathologically by biopsy, while biopsy can be avoided for HCC if the tumor shows typical imaging findings. Therefore, it is crucial to diagnose HCC accurately with imaging^3^.

Imaging findings are different between progressed- and early HCCs. On dynamic scans, progressed HCCs typically reveal hypervascularity in the hepatic arterial phase (HAP) followed by washout in the portal venous- or equilibrium phase (PVP, EP). Most early HCCs are hypovascular in the HAP. Consequently, to differentiate between early- and progressed HCC, the intra-tumoral vascularity in the HAP must be evaluated. However, conventional hepatic dynamic CT, three-phase (HAP, PVP, EP) contrast-enhanced CT acquired with single-energy CT, is less sensitive for the detection of HCC hypervascularity than other imaging modalities such as CT during hepatic arteriography (CTHA)^4^. Washout on PVP or EP images is suggestive of HCC rather than an arterioportal shunt. However, when the HCC is small, washout in the delayed phase is not always visible; this makes its differentiation from small arterial hypervascular lesions difficult^5,6^. Moreover, blood flow quantification helps to predict the HCC grade and to evaluate the treatment response to anti-angiogenic drugs^7^. Blood flow may be quantified with the change in the CT values because the concentration of contrast medium in blood vessel and tissue is linearly proportional to the increase in CT values. However, on conventional hepatic dynamic CT scans, accurate blood flow quantification based on changes in the CT value is difficult because factors other than the contrast medium also affect the value. Consequently, the diagnostic ability of conventional hepatic dynamic CT scans is not sufficient for the management of HCCs.

Dual-energy CT (DECT) facilitates material decomposition (e.g. iodine quantification) by acquiring two sets of images of the same body site by applying different photon spectra (high and low kV). The accurate estimation of contrast enhancement by iodine quantification may improve the diagnostic ability of dynamic CT in patients with HCC^7,8^. The utility of iodine maps derived from DECT for assessing the arterial vascularity of HCCs has been reported^9,10^. However, its utility for assessing arterial vascularity of HCCs based on a reference such as CTHA findings and also of washout in the EP remains to be determined.

Fast kilovolt-switching CT (FKSCT) scanner, one of the DECT platforms, uses a single X-ray tube that rapidly alternates between low and high energies. One major challenge in FKSCT imaging is that data-domain material decomposition is not readily available because raw data-based decomposition requires at least two different energy measurements for each view. As each view only has one energy measurement present for FKSCT scanning, interpolation techniques are needed to recover the missing view at each energy. However, this limits accuracy because full recovery of the missing view is difficult with interpolation techniques^11^. Deep learning-based spectral CT imaging (DL-SCTI) (Aquilion One Genesis, Canon Medical Systems, Otawara, Japan) is a novel type of FKSCT scanner that applies deep learning technology; two deep convolutional neural networks (DCNN) trained on fully sampled dual-energy data acquired via dual kV rotations are used. One of them completes the views missing in the sinogram space and the other improves the image quality in the image space^11,12^. This suggests that DL-SCTI may offer better energy separation than conventional FKSCT and facilitate robust material-decomposition analysis including iodine quantification^11,13^. We hypothesized that iodine quantification on DL-SCTI scans permits a more accurate assessment of HCC vascularity than the reference virtual monochromatic 70 keV images, thereby improving the ability to diagnose HCCs.

We compared the clinical utility of iodine maps generated from HAP and EP DL-SCTI scans with that of virtual monochromatic 70 keV images with respect to the quantification of the contrast uptake and the washout in HCCs.

## Materials and methods

The accuracy of iodine maps cannot be evaluated in clinical studies because the actual iodine concentration in the target area cannot be confirmed. Thus, our investigation included a phantom study where the iodine concentration is known for assessing the accuracy of iodine maps and a clinical study for the investigation of the clinical utility of iodine maps.

### Deep learning-based spectral CT imaging

Deep learning-based spectral CT imaging (DL-SCTI) is a single-source DECT scanner with fast kilovolt-switching and deep learning spectral reconstruction (Aquilion One Genesis, Canon Medical Systems, Otawara, Japan). The details of deep learning spectral reconstruction of DL-SCTI have been reported^11,12^. In summary, the entire processing pipeline consists of two DCNN trained on fully sampled dual-energy data acquired via dual kV rotations separated in two cascaded stages, one in the sinogram- and the other in the image space. The sparse low- and high-kV sinograms are first processed by the first DCNN so that the missing measurements are filled in. Then, the data-domain material decomposition process converts the fully-sampled low- and high kV projections into two basis materials, for example, iodine- and water projections, followed by separate analytic reconstructions. The reconstructed images are then processed by the second DCNN to improve the noise and resolution.

### Phantom study

To evaluate the accuracy of iodine maps derived from DL-SCTI scans we performed a phantom study. Our phantom harboring multiple cylindrical holes was scanned on a DL-SCTI. The acrylic phantom base was created with a 3D printer.

**Figure 1:**
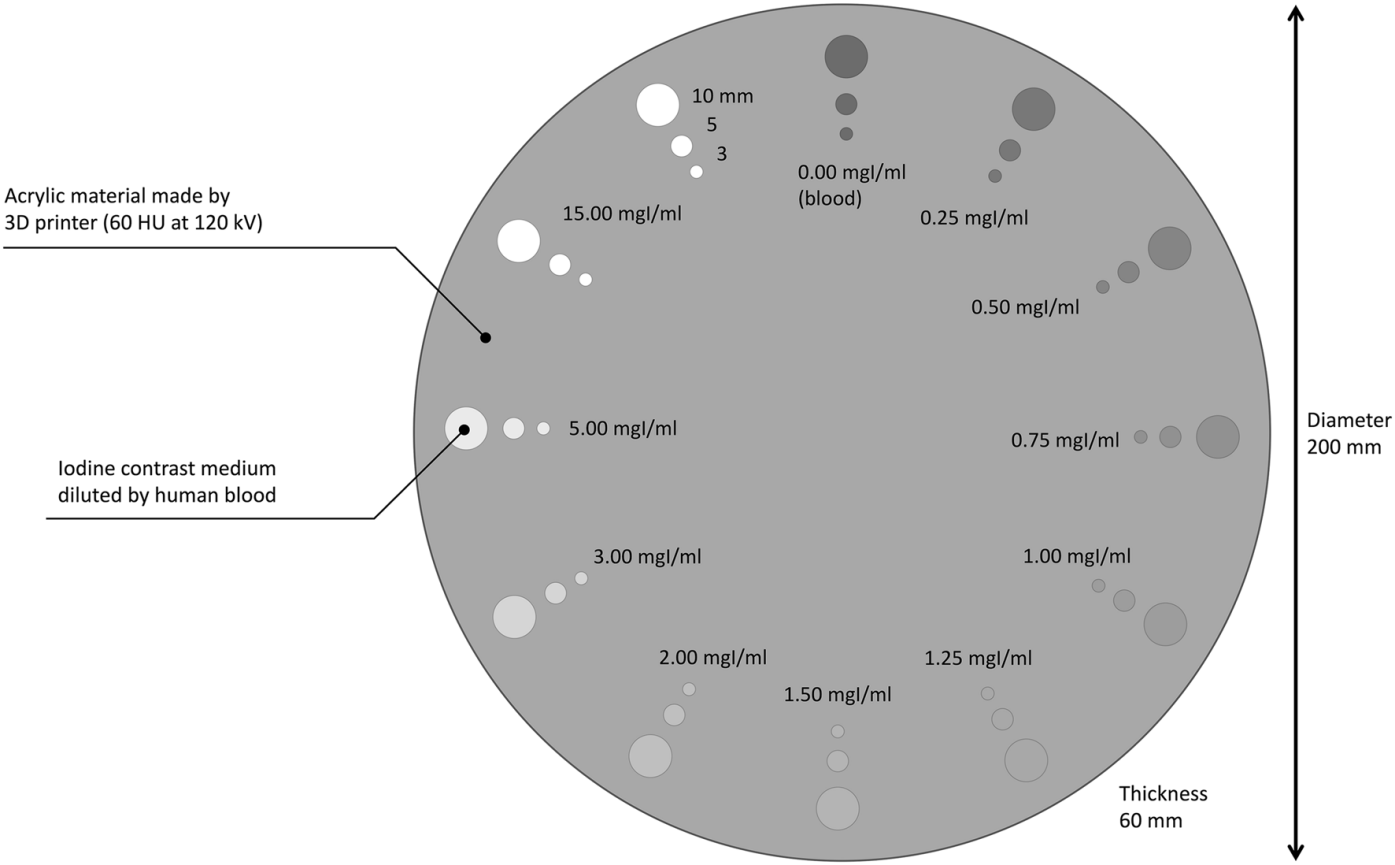
Design of the phantom.

**Figure 2:**
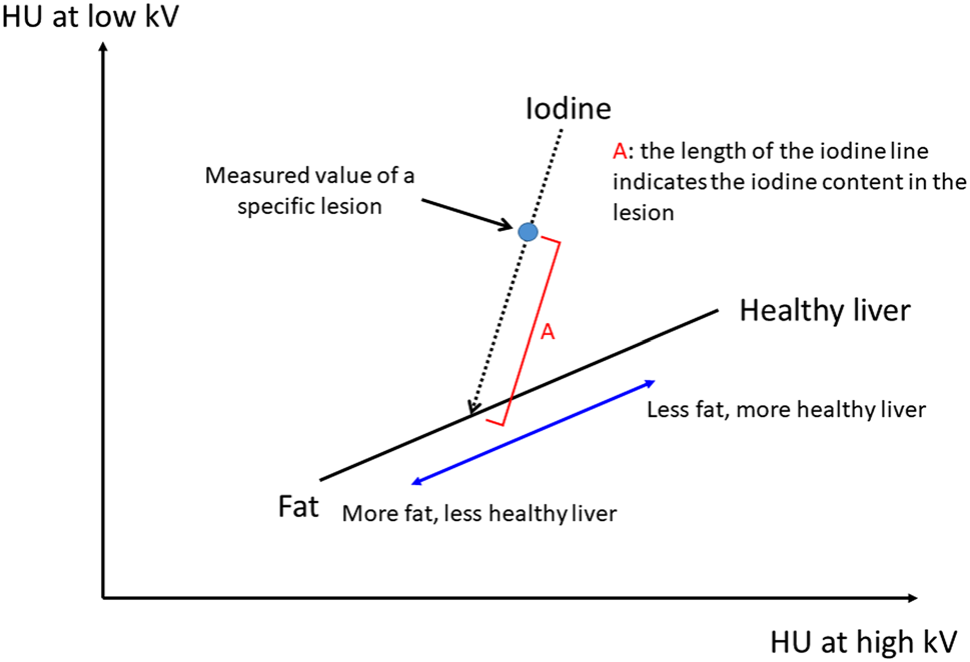
Diagram of three material decomposition.

As shown in Fig. 1, each hole (3-, 5-, or 10 mm in diameter with the length of 50 mm) was filled with 0.00-, 0.25-, 0.50-, 0.75-, 1.00-, 1.25-, 1.50-, 2.00-, 3.00-, 5.00-, or 15.00 mgI/ml contrast medium (CM) (Iohexole, Daiichi-Sankyo) diluted with commercially available human whole blood (Tennessee Blood Services, Memphis, TN, USA). The x-ray tube voltage was 135 and 80 kV; the x-ray tube current 300 mA, the CT dose index (CTDI)_vol_ 7.7 mGy, the detector configuration 0.5 × 80 mm rows, and the rotation time was 0.5 s/rotation. Using three-material decomposition (fat, healthy liver tissue, iodine), iodine maps were reconstructed using Spectral Analysis (Vitrea workstation (Version 7.14.2.227): Canon Medical Systems https://www.vitalimages.com/spectral-analysis/)^14–16^. A brief outline for the three-material decomposition (fat, healthy liver tissue, iodine) is as follows. Iodine leads to a very large difference between low- and high kV. The diagram (Fig. 2) shows the CT value at low- versus high kV images; iodine enhancement is represented by a vector of fixed direction whose length depends on iodine attenuation. In the diagram, typical body materials (fat, water, soft-, and liver tissue) are approximately located on a straight line. To obtain the iodine content a two-dimensional linear equation is solved. In the phantom study the iodine concentration was calculated as mgI/ml to compare it with the known iodine concentration.

#### Image analysis

Images were analyzed by one radiologist (SK with 6 years of experience in radiology) using ImageJ software (http://rsb.info.nih.gov/ij/). Regions of interest (ROIs) were placed on each hole and the mean value inside each ROI was recorded.

### Clinical study

This retrospective, observational study was approved and the need for informed consent was waived by the Ethical Committee for Epidemiology of Hiroshima University. Patient records and information were anonymized and de-identified prior to analysis. The study was performed in accordance with our institution’s relevant guidelines and regulations.

#### Study population

The inclusion criteria for consecutive 62 HCC patients who were seen between July 2019 and December 2020 were as follows: (a) the HCC diagnosis was based on pathologic proof of the tumor burden obtained after partial hepatectomy, (b) patients who underwent preoperative hepatic dynamic CT acquired with DL-SCTI plus CTHA studies. Hypervascular HCCs should be evaluated accurately because they are much more aggressive than hypovascular HCCs^4,17^. Raw data of DL-SCTI scanning is required for reconstruction of iodine maps. Thus, the exclusion criteria were as follows: (a) patients with HCCs which were hypovascular, (b) raw data of DL-SCTI scanning was not stored. Tumor vascularity was confirmed with CTHA. One board-certified radiologist (KN with 7 years of experience in radiology) placed ROIs on the tumor and the surrounding hepatic parenchyma and calculated the contrast ratio (CR) on CTHA images as CR = ROI_T_/ROI_L_, where ROI_T_ is the mean attenuation of the tumor, and ROI_L_ the mean attenuation of the liver parenchyma. HCCs with a CR > 1.0 were defined as hypervascular^18^. KN also confirmed the presence and location of hypervascular HCCs on dynamic CT images. Finally, this study evaluated 52 patients with hypervascular HCCs (37 men, 15 women; age range 50–88 years, median age 70.0 years). All 52 patients had chronic liver disease and its underlying causes were hepatitis B (n = 15), hepatitis C (n = 24), alcoholic chronic hepatitis (n = 3), nonalcoholic steato-hepatitis (n = 6), autoimmune hepatitis (n = 1), primary biliary cirrhosis (n = 1), and unknown (n = 2).

#### Image acquisition

All CT images were scanned with DL-SCTI. The scanning parameters were rotation time 0.5 s, beam collimation 80 × 0.5 mm, section thickness and interval 5.0 mm, pitch factor 0.637, table movement 50.96 mm/s, scanning field of view (FOV) 40 cm, voltage 135 and 80 kV, and volume EC [noise index 10 Hounsfield units (HU)]. Volume EC is an automatic tube-current modulation technique that maintains the image quality over a range of patient sizes by automatically adjusting the tube current to the x-ray attenuation of the section being scanned^19^.

Three-phase dynamic CT images were obtained during the HAP, PVP, and EP according to a previous paper^18^. Automatic bolus-tracking program was used to time the start of scanning for each phase. The trigger threshold level was set at 200 HU in the abdominal aorta at the L1 vertebral body level. HAP, PVP, and EP scans were started at 17-, 47-, and 152 s, respectively. The CM dose in all patients was 600 mgI/kg; it was delivered with a power injector and a 20-gauge intravenous catheter inserted into an antecubital vein. The injection duration was 30 s. CM delivery was followed by flushing with 30 ml of physiologic saline administered at the same injection rate. Virtual monochromatic images (VMIs) at 70 keV were reconstructed as the reference images because the standard of care in our institution for abdominal DECT includes 70 keV monoenergetic images as a standard reconstruction method for image evaluation compared to routine 120 kV single-energy CT^20–22^. A brief outline for generating VMIs is as follows. The CT value at arbitrary energy levels can be estimated with the mass density and linear attenuation coefficient of water and iodine. As the linear attenuation coefficient of iodine and water at arbitrary energy levels is known, their mass density can be calculated using each CT value acquired at two energy levels. Therefore, simulated monochromatic CT images at arbitrary energy, VMIs, can be generated with dual-energy CT. With three-material decomposition (fat, healthy liver tissue, iodine) we also reconstructed iodine maps for both the HAP and EP^14–16^. The 70 keV images and the iodine maps were reconstructed using Spectral Analysis (Vitrea workstation (Version 7.14.2.227): Canon Medical Systems https://www.vitalimages.com/spectral-analysis/). As HU is the most commonly used unit for CT images, the degree of contrast enhancement can be understood easily when it is expressed in HU especially for clinical study. In addition, the 70 keV images are expressed in HU. Therefore, the iodine concentration in the clinical study is also expressed in HUs rather than mgI/ml. Although PVP scans were obtained we evaluated only HAP and EP images.

CTHA was performed using a 16 detector-row interventional radiology scanner based on a previous paper^23^. After femoral artery puncture, a 4-Fr catheter was selectively placed in the common or proper hepatic artery. Images were acquired throughout the liver in a craniocaudal direction. The CT imaging parameters were rotation time 0.5 s, beam collimation 16 × 1 mm, section thickness and interval 5.0 mm, pitch factor 1.313, scanning FOV 40 cm, voltage 120 kV, and volume EC (noise index 11 HU). In patients with no anatomic variation of the hepatic artery, CTHA scanning began 10 s after starting the infusion of CM (24 ml diluted with 48 ml of physiologic saline, iodine concentration 350 mgI/ml) into the common or proper hepatic artery at a rate of 1.5 ml/s. In patients with anatomic variation (right hepatic artery branching from the superior mesenteric artery or the common hepatic artery, left hepatic artery branching from the left gastric artery), CTHA was performed via the right and left hepatic artery. We infused the CM (12 ml diluted with 24 ml of physiologic saline, iodine concentration 350 mgI/ml) at 1.0 ml/s into each artery. All patients underwent double-phase CTHA; scanning began 10 and 30 s after the start of the infusion. The CM was delivered via a power injector.

#### Image analysis

Each patient was considered to be a cluster. Observations within the same cluster were correlated. As conventional statistical techniques are based on the assumption that all observations are independent of each other, in patients with multiple lesions only one lesion should be evaluated to avoid the statistical clustering effect^24^. Because it is more difficult to detect small- than large tumors, the ability to visualize small tumors is of great clinical importance and requires improvement. In addition, on dynamic images, larger HCCs show typical enhancement patterns while the patterns are atypical in the presence of small HCCs, suggesting that a special technique is needed in patients with small HCCs^25^. Therefore, in patients with multiple HCCs we only evaluated the smallest tumor.

One radiologist (KN) subjected 70 keV images and iodine maps acquired during the HAP and EP to quantitative image analysis. To measure attenuation, an ROI was placed on the tumor, the adjacent hepatic parenchyma, and the paraspinal muscle. Each measurement was repeated three times. The standard deviation (SD) of the paraspinal muscle was used as the image noise^26^.

Compared with the surrounding hepatic parenchyma, typical hypervascular HCCs exhibit high density during the HAP and low density during the EP. The contrast during the HAP (contrast_a_) was calculated as contrast = (ROI_T_—ROI_L_), the contrast during the EP (contrast_e_) as contrast = (ROI_L_—ROI_T_), the contrast-to-noise ratio (CNR) at the HAP (CNR_a_) as contrast_a_/N, and the CNR during the EP (CNR_e_) as contrast_e_/N. ROI_L_, indicates the mean attenuation of the hepatic parenchyma, ROI_T_ the mean attenuation of the tumor, and N the noise^26^.

For qualitative image analysis, during the HAP and EP, each liver lesion on 70 keV images and on iodine maps was reviewed independently by two other board-certified radiologists (YN and KA with 16 and 33 years of experience in radiology, respectively). They scored tumor visibility vis-à-vis the surrounding hepatic parenchyma on a 5-point confidence scale (Likert scale) where 1 = poor tumor contrast, tumor not identifiable, 2 = reduced tumor contrast, tumor not visible, 3 = reduced tumor contrast, tumor detection possible, 4 = good tumor contrast, not all tumor parts identifiable, and 5 = excellent tumor contrast, tumor easily detectable^27–29^. In the absence of interobserver agreement, final decisions were reached by consensus. We defined lesions with score of 3 or higher as lesions detected by qualitative assessment and lesions with score of 2 or lower as undetectable.

#### Radiation dose

To assess radiation exposure, the CTDI_vol_ and the dose-length product (DLP) displayed on the scanner console were recorded. The size-specific dose estimate (SSDE), an index in which the CTDI is corrected by the body habitus, was also calculated^30,31^. Size-dependent conversion factors were obtained from AAPM Report 204^32^; they were based on the sum of the antero-posterior and lateral dimensions at the mid-liver level of each patient.

### Statistical analysis

Statistically significant differences were evaluated with JMP Pro 15 software (SAS Institute). To determine the difference between the estimated iodine concentration on iodine maps and the known concentrations in the phantom study, the mean, standard deviations, and the mean squared errors of the difference were calculated. The mean squared error, the average of the square of the errors, closer to 0 indicates a smaller difference between the estimated- and the known iodine concentration. The relative squared error is the squared error value divided by the known iodine concentration. CNR differences between the 70 keV images and the iodine maps were determined using the two-sided Wilcoxon signed-rank test. We used the McNemar test for qualitative analysis. Differences of *p* < 0.05 were considered statistically significant.

For qualitative analysis we calculated the interobserver agreement using the weighted kappa statistic to evaluate agreement between the two readers. A kappa statistic in the range of 0.81–1.00 was interpreted as excellent-, 0.61–0.80 as substantial-, 0.41–0.60 as moderate-, 0.21–0.40 as fair-, and 0.00–0.20 as poor agreement^33^.

## Results

### Phantom study

**Figure 3:**
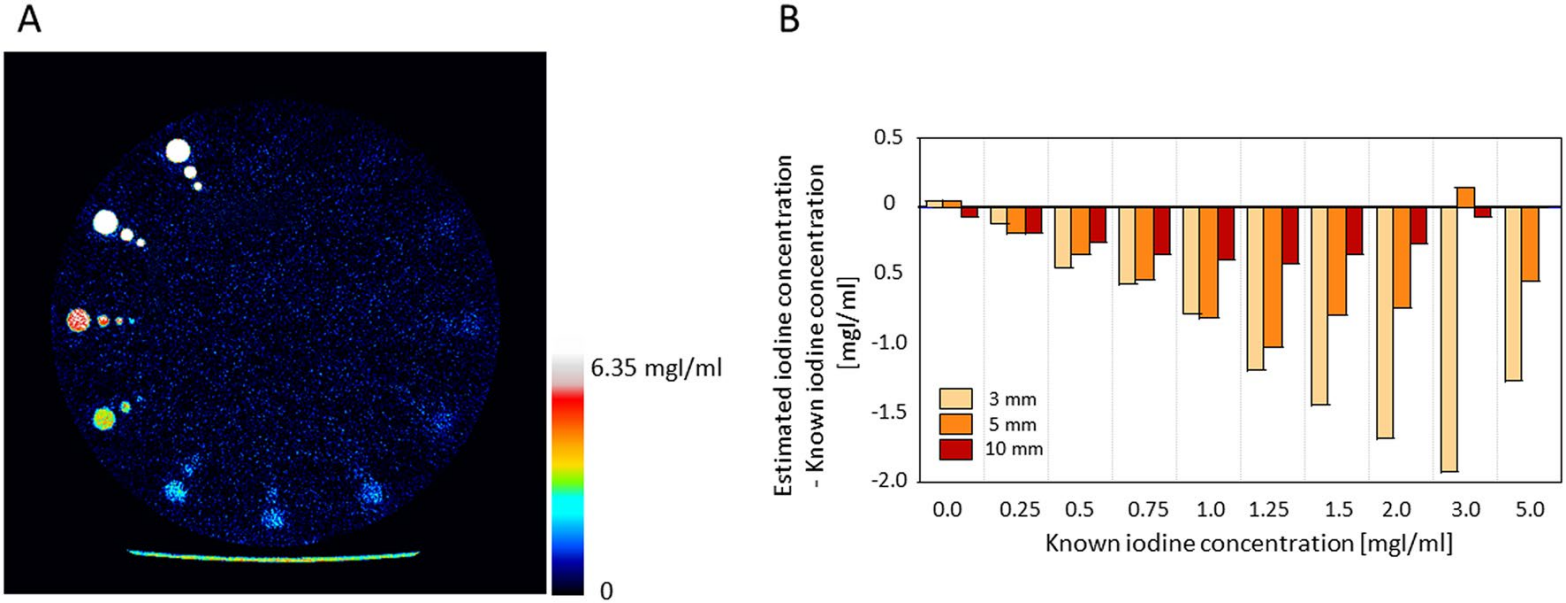
(**A**) Iodine map of the phantom. (**B**) The difference between the estimated- and the known iodine concentration. High-concentration modules were clearly visualized. Low-concentration modules, especially those with an iodine concentration below 0.75 mgI/ml and a diameter of 10 mm, and 3-mm modules with an iodine concentration below 1.5 mgI/ml were not because the background noise was high. The difference between the estimated iodine concentration generated from iodine maps and the known iodine concentration was small in 10-mm modules. The estimated iodine concentration was underestimated especially in modules with the smallest diameter (3 mm). There was some underestimation even in 10-mm modules whose concentration was < 2.0 mgI/ml. Iodine maps were generated using Spectral Analysis (Vitrea workstation (Version 7.14.2.227): Canon Medical Systems https://www.vitalimages.com/spectral-analysis/).

Figure 3A is an iodine map derived from DL-SCTI scans. The high-concentration modules of all diameters were clearly visualized. Due to background noise, the low-concentration modules, especially those with an iodine concentration below 0.75 mgI/ml and a diameter of 10 mm, and 3-mm modules with a concentration of 1.5 mgI/ml were not visualized.

Figure 3B shows the difference in the results of converting the iodine map into an estimated iodine- and the known iodine concentration value. On iodine maps, the mean (SD) and the mean squared error (mean relative squared error) of the difference between the estimated- and the known iodine concentration was − 0.06 (0.59) and 0.07 (0.01), − 0.47 (0.36) and 0.36 (0.12), and − 1.01 (1.22) and 1.27 (0.63) mgI/ml, respectively, for 10-, 5-, and 3-mm modules (Table 1), indicating that the estimated iodine concentration generated from iodine maps was underestimated especially for small diameter modules. There was some underestimation even for 10-mm, low-concentration modules, especially when the iodine concentration was below 2.0 mgI/ml.

**Table 1:** Squared- and relative squared errors of the difference between the estimated concentration on iodine maps and the known iodine concentration.

Known iodine concentration (mgI/ml)	Squared error			Relative squared error		
	10 mm	5 mm	3 mm	10 mm	5 mm	3 mm
5.00	0.00	0.28	1.60	0.00	0.09	0.80
3.00	0.01	0.02	3.69	0.00	0.01	1.84
2.00	0.07	0.54	2.83	0.01	0.18	1.41
1.5	0.12	0.61	2.06	0.02	0.20	1.03
1.25	0.17	1.03	1.39	0.03	0.34	0.69
1.00	0.15	0.65	0.60	0.03	0.22	0.30
0.75	0.12	0.28	0.32	0.02	0.09	0.16
0.5	0.07	0.11	0.19	0.01	0.04	0.10
0.25	0.04	0.03	0.01	0.01	0.11	0.01
0.00	0.00	0.00	0.00	0.00	0.00	0.00

### Clinical study

The median diameter of the smallest tumor in each patient was 13.6 mm (range 6.5–92.0 mm). Of the 52 HCCs 12 were well, 39 were moderately, and 1 was poorly differentiated. The average ROI size for the tumors was 478.7 ± 1187.5 mm^2^ (SD) (range 42.6–8465.4 mm^2^); it was 435.3 ± 231.4 mm^2^ (SD) (range 83.2–1047.9 mm^2^) for the hepatic parenchyma, and 250.9 ± 87.5 mm^2^ (SD) (range 67.4–465.5 mm^2^) for the paraspinal muscle. On both the 70 keV images and the iodine maps, the contrast was higher in the HAP than the EP (Table 2). On 70 keV images, the median contrast value was less than 10 HU during the EP.

**Table 2:** Image noise, contrast, and CNR on 70 keV images and iodine maps.

	70 keV	Iodine map	P value
HAP			
Noise (HU)	7.60 (4.26–28.74)	3.90 (1.44–10.28)	
Contrast_a_ (HU)	25.56 (− 31.18 to 99.12)	23.61 (− 3.42 to 87.68)	
CNR_a_	2.65 (− 1.10 to 14.47)	6.61 (− 0.78 to 26.21)	< 0.01
EP			
Noise (HU)	7.21 (3.75–31.96)	4.59 (1.77–7.00)	
Contrast_e_ (HU)	6.69 (− 6.70 to 36.1)	− 1.59 (− 19.99 to 13.98)	
CNR_e_	0.72 (− 0.72 to 9.63)	− 0.33 (− 7.14 to 3.03)	< 0.01

Data are the median with ranges in parentheses.

HAP: Hepatic arterial phase, EP: equilibrium phase. Contrast_a_: contrast at HAP, Contrast_e_: contrast at EP. CNR_a_: contrast-to-noise ratio at HAP, CNR_e_: contrast-to-noise ratio at EP, HU: Hounsfield units.

**Figure 4:**
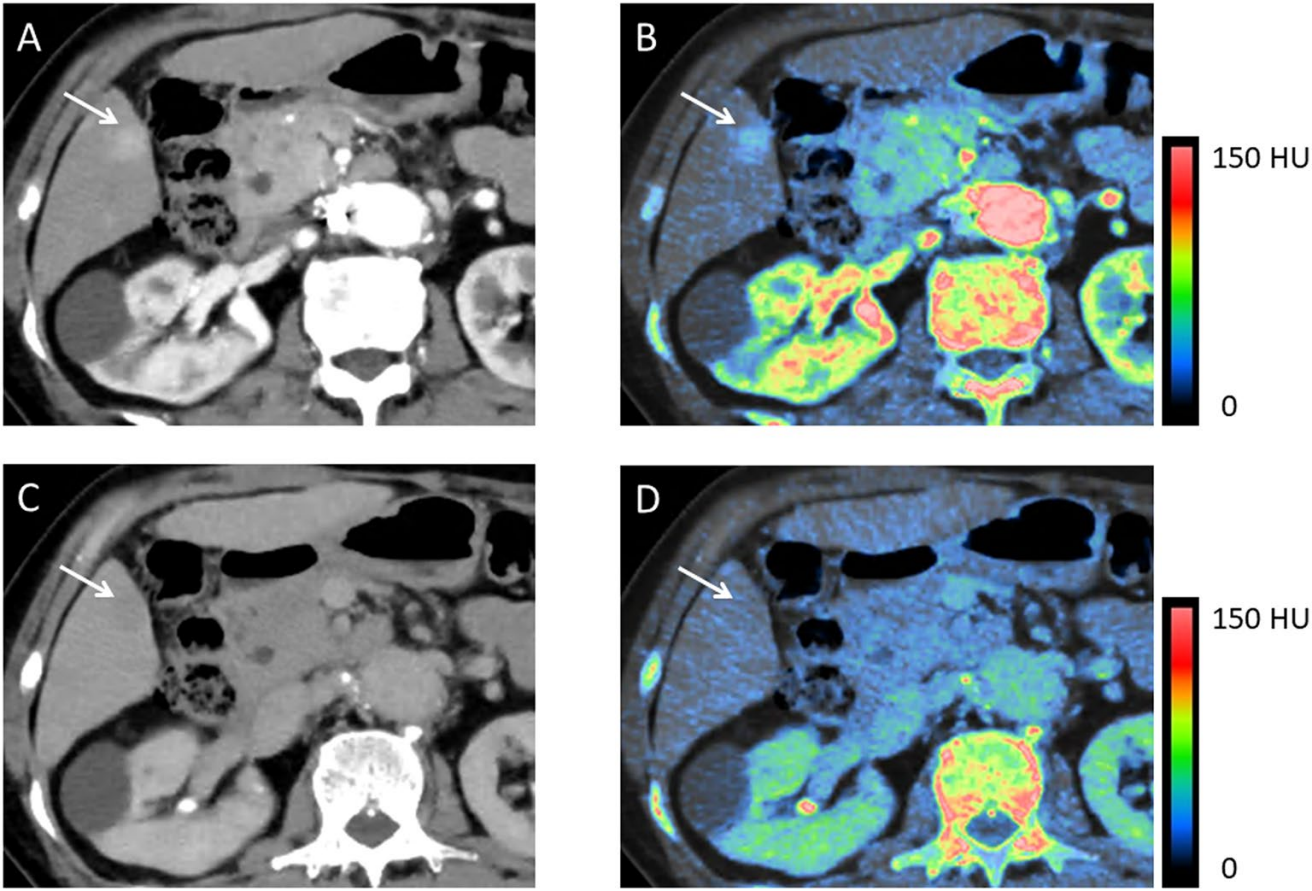
Hypervascular HCC in a 76-year-old woman. (**A**) 70 keV image (HAP). (**B**) Iodine map (HAP). (**C**) 70 keV image (EP). (**D**) Iodine map (EP). During the HAP, the HCC (arrow) was more clearly visualized on the iodine map (**B**) than the 70 keV image (**A**). During the EP, the HCC washout (arrow) was demonstrated on the 70 keV image (**C**) but not on the iodine map (**D**). 70 keV images and iodine maps were generated using Spectral Analysis (Vitrea workstation (Version 7.14.2.227): Canon Medical Systems https://www.vitalimages.com/spectral-analysis/).

**Figure 5:**
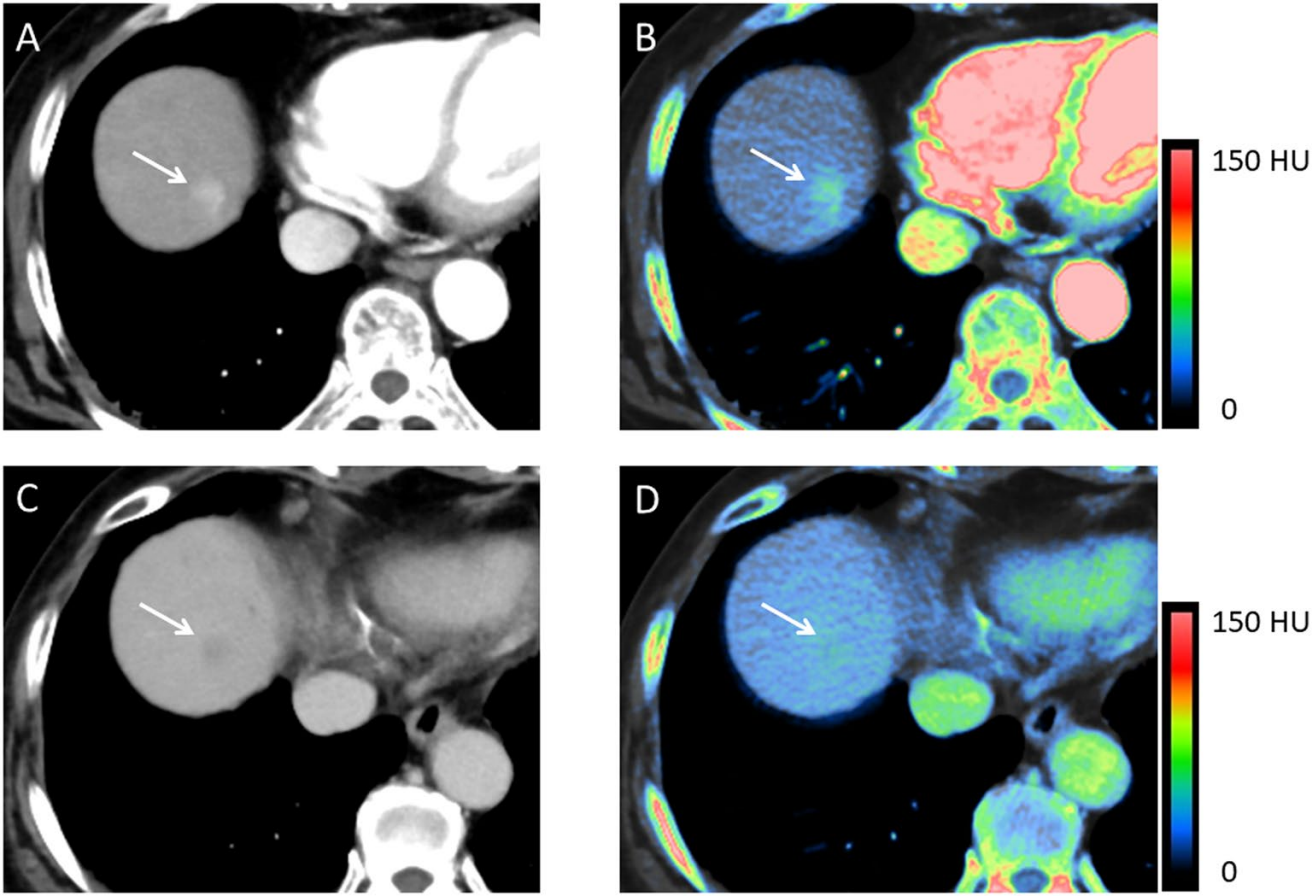
Hypervascular HCC in a 72-year-old woman. (**A**) 70 keV image (HAP). (**B**) Iodine map (HAP). (**C**) 70 keV image (EP). (**D**) Iodine map (EP). During the HAP, visibility of the HCC (arrow) was almost equivalent to the iodine map (**B**) and the 70 keV image (**A**). In the EP, the tumor (arrow) was visualized on the 70 keV image (**C**), but not on the iodine map (**D**).

**Figure 6:**
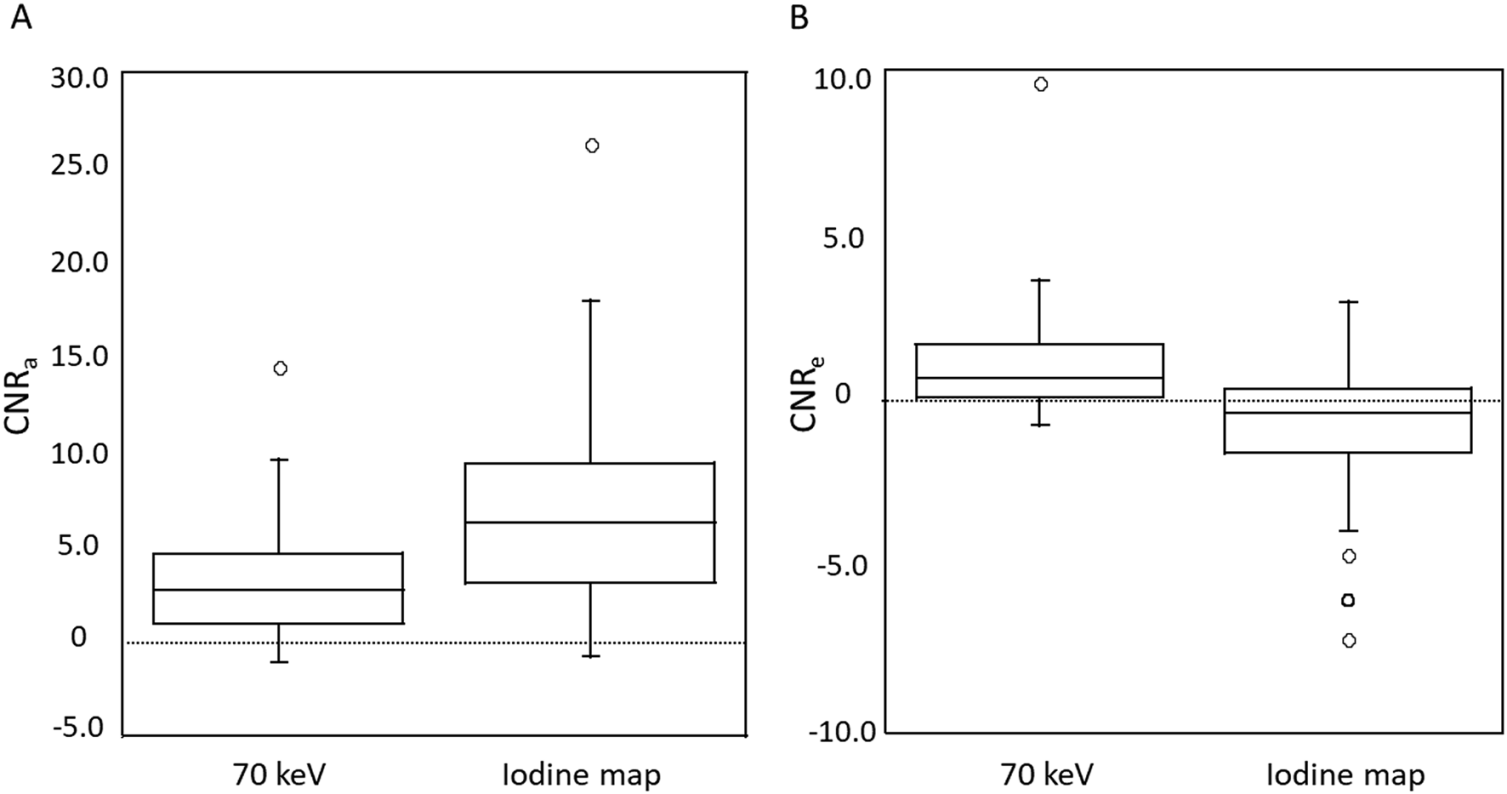
Plot showing the CNR of HCCs on 70 keV images and iodine maps during the hepatic arterial phase (CNR_a_) (**A**) and the equilibrium phase (CNR_e_) (**B**). The vertical line indicates the median CNR. Only one HCC had a negative CNR_a_ on iodine maps; 5 had a negative CNR_a_ on 70 keV images. Of the 52 tumors, 22 had a positive CNR_e_ (washout) on iodine maps; 41 had a positive CNR_e_ on 70 keV images. 70 keV images and iodine maps were generated using Spectral Analysis (Vitrea workstation (Version 7.14.2.227): Canon Medical Systems https://www.vitalimages.com/spectral-analysis/).

The CNR_a_ was significantly higher on iodine maps than on 70 keV images (*p* < 0.01) (Table 2 and Fig. 4). Only one of 52 HCCs had a negative CNR_a_ on iodine maps; on 70 keV images 5 had a negative CNR_a_ (Fig. 6A). The CNR_e_ was significantly higher on 70 keV images than on iodine maps (*p* < 0.01) (Table 2). Fewer HCCs had a positive CNR_e_ (washout) on iodine maps (n = 22) than on 70 keV images (n = 41) (Figs. 4, 5 and 6B).

On HAP iodine maps, 96.1% of lesions were assigned a confidence score of 3 or higher; they were detected by qualitative assessment; 94.2% were detected on 70 keV images (*p* < 0.01) (Table 3 and Fig. 4). On EP iodine maps, 46.2% of the lesions were detected, on 70 keV images 69.2% were identified (*p* < 0.01) (Table 4, Figs. 4 and 5). Interobserver agreement between the two readers was substantial (kappa value range 0.70–0.75).

**Table 3:** Subjective image quality scores for HCCs during the hepatic arterial phase on 70 keV images and iodine maps.

Score	70 keV	Iodine map
1	0	0
2	3	2
3	24	16
4	18	17
5	7	17

**Table 4:** Subjective image quality scores for HCCs during the equilibrium phase on 70 keV images and iodine maps.

Score	70 keV	Iodine map
1	9	15
2	7	13
3	16	10
4	14	9
5	6	5

On CT images of each phase, the median CTDI_vol_ was 13.4 mGy (range 8.9–18.6), the median DLP was 333.5 mGy cm (range 206.6–535.7), and the median SSDE was 19.4 mGy (range 15.2–21.8). These values were lower than on conventional hepatic dynamic CT scans promulgated as the Japanese diagnostic reference levels^34^.

## Discussion

Our clinical study showed that during the HAP, the CNR_a_ and the number of qualitatively detected HCC lesions was significantly higher on iodine maps than on 70 keV images. On conventional contrast-enhanced images the tumor-to-liver contrast corresponds with the difference in attenuation but not with the degree of enhancement^35^. In our phantom study, the estimated iodine concentration derived from DL-SCTI scans was highly correlated with the known iodine concentration in 10-mm modules. We therefore inferred that a higher CNR_a_ was obtainable on iodine maps than on 70 keV images because the tumor-to-liver contrast on iodine maps corresponds with the degree of enhancement. Consequently, we suggest that iodine maps generated from DL-SCTI scans are superior to conventional HAP images such as 70 keV images for the evaluation of HCC hypervascularity. Although the utility of iodine maps derived from DECT for assessing the arterial vascularity of HCCs has been reported^9,10^, we suggest that the robustness of our findings is superior because we referred to CTHA images to evaluate arterial vascularity.

Not all of our HCCs had a positive CNR_a_ on HAP iodine maps although its rate was higher than on 70 keV images. In our phantom study, the estimated iodine concentration generated from iodine maps was underestimated especially in small-diameter modules and in large-diameter modules with an iodine concentration of less than 2.0 mgI/ml. This indicates that slight enhancement, especially of small lesions, may not be detected on iodine maps. The reasons for underestimation of low iodine concentration are considered as follows. At DECT iodine concentration is calculated based on the difference in the attenuation coefficients of iodine obtained at two different energies. Therefore, when the concentration of iodine is low the difference in the attenuation coefficients obtained at two different energies between iodine and other material is small, resulting in the difficulty of distinguishing iodine from other materials. The background noise also hampers the detectability of small- and low-concentration modules because the change derived from small- and low-concentration modules on iodine maps is small. The accuracy of iodine quantification in small-diameter- and low-concentration (below 2 mgI/ml) modules was not confirmed in a study that used other DECT implementations^36^. This issue is supposed to be general limitations of DECT and requires attention. The quality of iodine maps derived from DECT must be improved to help in the evaluation of HCC vascularity.

The CNR_e_ of HCCs and the rate of qualitatively detected lesions was significantly lower on EP iodine maps than on 70 keV images and fewer HCCs had a positive CNR_e_ (washout) on iodine maps than on 70 keV images. CM is administered to increase the inherent contrast differences between the liver and the tumor, and to do this successfully, the CM must predominately reach the normal liver or the focal liver lesion, but not both^35^. On unenhanced images, the density of HCCs tends to be lower than of the surrounding hepatic parenchyma. When approximately the same amount of CM enters both the HCC and the liver parenchyma, the tumor is low-dense on conventional contrast-enhanced images, including 70 keV images; it is isodense on iodine maps due to the correspondence with the degree of enhancement. On EP 70 keV images, the median contrast value was less than 10 HU and almost equal to an iodine concentration of 0.4 mgI/ml^37^. Consequently, slight enhancement differences may not be detectable on iodine maps due to the background noise and underestimation of the iodine concentration. Our observations lead us to suspect that iodine maps generated from DL-SCTI scans do not improve the identification of washout in the EP.

Our retrospective, single-center study has some limitations. As the number of HCCs was relatively small, our findings are preliminary. Because DECT involves a second radiation exposure that increases the delivered radiation dose; this issue required further investigation^38^. Nonetheless, the radiation dose was lower in our study than the dose delivered at conventional hepatic dynamic CT promulgated as the Japanese diagnostic reference level^34^. We evaluated EP- rather than PVP images in our evaluation of HCC washout because characterizing lesions on PVP- in the absence of EP images can result in HCC downgrading^39^. PVP imaging is important for the evaluation of hypovascular hepatic metastases and of abnormalities in the portal venous system^40^. Further studies that include PVP images and are focused on the evaluation of hepatic metastases are needed.

The ideal design for evaluating utility is a comparison of DL-SCTI with conventional single-energy CT. However, the simultaneous acquisition of DL-SCTI- and single-energy CT scans exposes patients to excessive radiation doses. Another way is to compare two patient groups where one group undergoes conventional single-energy CT scanning and the other DL-SCTI. However, because HCCs yield variable imaging findings, including with respect to their vascularity, it is not easy to match separate patient groups based on their background and HCC imaging findings. Therefore, instead of single-energy CT images we used 70 keV VMIs of the same patient as a reference. VMIs at multiple energy levels can be generated from DECT scans. As low keV-level monoenergetic images are characterized by higher contrast resulting from the maximum iodine attenuation that is close to the K edge of iodine (33.2 keV), this improves the conspicuity of hepatic lesions, including HCCs^41^. However, as the image noise increases on VMI datasets acquired below 70 keV^42^, we used only 70 keV images for standard reconstruction^21,22^. Not available at our institute is the advanced noise-optimized virtual monoenergetic reconstruction algorithm that combines the greater iodine attenuation of the standard VMI algorithm at low virtual photon energies with the lower image noise at higher virtual photon energies. It adds a dual-layer detector system, another type of DECT platform that yields a higher CNR at low virtual photon energies^43,44^. The utility of low (40–50 keV) keV noise-optimized VMIs for the accurate assessment of HCC vascularity should be verified. Lastly, we did not compare DL-SCTI and other DECT implementations in this single-center study. As scanning- and analysis methods for iodine quantification at DECT vary by the instrument vendor, the ability of DECT with and without deep leaning should be compared on scanners from the same vendor to evaluate the effect of deep learning technology. However, our clinical DL-SCTI scanner does not allow scanning and analysis without deep learning technology. According to Zhang et al.^11^, the addition of deep-learning technology improved the accuracy of iodine quantification. Therefore, unlike other DECT scanners, DL-SCTI with deep learning may yield robust iodine quantification results. However, more studies are needed to compare the clinical utility of DL-SCTI and DECT scanners.

In conclusion, iodine maps generated from DL-SCTI scans are useful for the assessment of arterial HCC vascularity. However, conventional contrast-enhanced images, including 70 keV images, are required to assess washout in the EP because iodine maps fail to address this issue. Also, when the lesion is small or the iodine concentration is low, iodine quantification may result in underestimation.

## Data Availability

The datasets used and/or analysed during the current study available from the corresponding author on reasonable request.

## Abbreviations

CT: Computed tomography
MRI: Magnetic resonance imaging
HCC: Hepatocellular carcinoma
HAP: Hepatic arterial phase
PVP: Portal venous phase
EP: Equilibrium phase
CTHA: Computed tomography during hepatic arteriography
DECT: Dual-energy CT
FKSCT: Fast kilovolt-switching CT
DL-SCTI: Deep learning-based spectral CT imaging
DCNN: Deep convolutional neural network
CM: Contrast medium
CTDI: CT dose index
ROI: Region of interest
CR: Contrast ratio
FOV: Field of view
HU: Hounsfield units
SD: Standard deviation
CNR: Contrast-to-noise ratio
DLP: Dose-length product
SSDE: Size-specific dose estimate
VMI: Virtual monochromatic image
